# Two-Dimensional Electrophoresis of Tau Mutants Reveals Specific Phosphorylation Pattern Likely Linked to Early Tau Conformational Changes

**DOI:** 10.1371/journal.pone.0004843

**Published:** 2009-03-17

**Authors:** Alexis Bretteville, Kunie Ando, Antoine Ghestem, Anne Loyens, Séverine Bégard, Jean-Claude Beauvillain, Nicolas Sergeant, Malika Hamdane, Luc Buée

**Affiliations:** 1 Inserm, U837, Place de Verdun, Lille, France; 2 Université Lille 2, Faculté de Médecine, Institut de Médecine Prédictive et Recherche Thérapeutique, Jean-Pierre Aubert Research Centre, Lille, France; Vrije Universiteit Medical Centre, Netherlands

## Abstract

The role of Tau phosphorylation in neurofibrillary degeneration linked to Alzheimer's disease remains to be established. While transgenic mice based on FTDP-17 Tau mutations recapitulate hallmarks of neurofibrillary degeneration, cell models could be helpful for exploratory studies on molecular mechanisms underlying Tau pathology. Here, “human neuronal cell lines” overexpressing Wild Type or mutated Tau were established. Two-dimensional electrophoresis highlights that mutated Tau displayed a specific phosphorylation pattern, which occurs in parallel to the formation of Tau clusters as visualized by electron microscopy. In fact, this pattern is also displayed before Tau pathology onset in a well established mouse model relevant to Tau aggregation in Alzheimer's disease. This study suggests first that pathological Tau mutations may change the distribution of phosphate groups. Secondly, it is possible that this molecular event could be one of the first Tau modifications in the neurofibrillary degenerative process, as this phenomenon appears prior to Tau pathology in an *in vivo* model and is linked to early steps of Tau nucleation in Tau mutants cell lines. Such cell lines consist in suitable and evolving models to investigate additional factors involved in molecular pathways leading to whole Tau aggregation.

## Introduction

Tau (tubulin associated unit) is a microtubule-associated protein. In the human brain, there are six Tau isoforms generated by alternative splicing. They differ by the combination of 0, 1 or 2 amino-terminal inserts and 3- or 4-microtubule-binding repeats (3R or 4R) encoded by exons 2,3 and 10 respectively. Tau aggregation is one of the key features common to Tauopathies, a group of neurodegenerative diseases including Alzheimer's disease (AD). Even though Tau is always found aggregated and hyperphosphorylated in these pathologies, the precise role of phosphorylation in Tau aggregation process is still debated. In the same way, physiopathological significance of Tau aggregation remains to be established. The discovery of Tau mutations associated with Frontotemporal Dementia with Parkinsonism linked to chromosome 17 (FTDP-17), has allowed for generating several animal models and especially Tau transgenic mice that display a Tau pathology characterized by abnormal phosphorylation and Tau aggregation [1]–[6]; and for review [7]. Beside these *in vivo* models, many attempts have been done to generate cell systems, which could recapitulate molecular features of Tau pathology and then could be more appropriate to carry exploratory studies on events involved in Tau aggregation and its role in neuronal death. Two studies with specific Tau constructs showed an abnormal Tau behaviour in cells. The first study based on overexpression of N-terminal half truncated Tau bearing ΔK280 mutation showed an increase in Tau aggregation [8]. The second one showed that breaking specific motifs in microtubule binding repeats [9] rapidly induce Tau aggregation and an appearance of phosphoepitopes observed in AD-Tau pathology. These models are interesting to give some insights into relationship between Tau structure and its aggregation but it is not clear that full-length Tau without these additional mutations follows the same process of aggregation. Indeed, several strategies based on either pharmacological treatments with okadaic acid and Hydroxy-nonenal [10] or overexpression of Tau bearing FTDP-17 mutations have been developed (for review [11]). Most of these models with full-length Tau fail to identify early molecular hallmarks of AD-Tau pathology. As almost of these studies have been done in either non-human cells or in “non-neuronal” human cells, the lack of Tau pathological features could be explained by differences in molecular contents between neuronal and non-neuronal cells. In the present work, using differentiated human neuroblastoma cell lines, both wild type and mutated Tau proteins were analyzed by a proteomic approach to evaluate the potential phosphorylation role in tau aggregation process.

## Results

### Characterisation of SH-SY5Y over-expressing 4RTau

**Figure 1:**
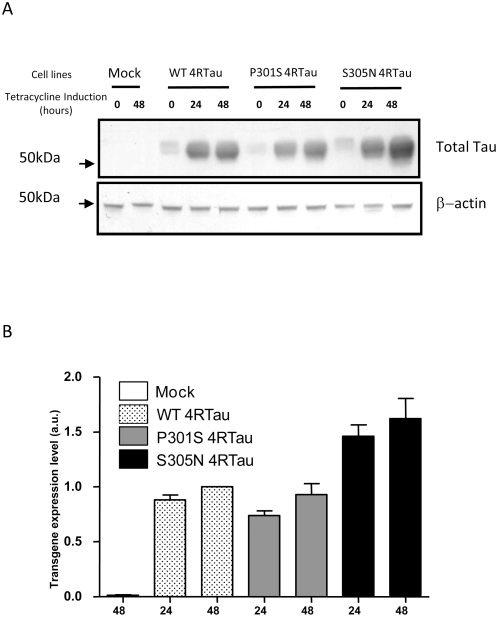
Analysis of transgenes expression in 4RTau cell lines. A) Lysates from Mock and 4RTau cells, treated or not (0) with tetracycline for 24 to 48 hours were immunolabeled with TauCter 1902 (Total Tau), and β-actin as loading control. B) Quantification of Tau expression levels in 4RTau cell lines: Ratios of densitometric values of TauCter1902/β-actin immunoreactivities are presented. Ratios are normalized to those obtained from *WT* 4RTau cells after 48 hours tetracycline (arbitrary value = 1).

In previous studies, we showed that, compared to 3R Tau, constitutive over-expression of 4R Tau increased susceptibility of SH-SY5Y neuroblastoma cells to cell death [12]. In order to avoid 4RTau toxicity and any interference with SY5Y differentiation, stable cell lines were established using an inducible system. As shown in Fig. 1A, endogenous Tau immunoreactivity was not observed at low exposure. In non-induced 4RTau cell lines, a low basal expression of exogenous Tau proteins due to a leak of the inducible expression system was observed. After tetracycline induction, a 4RTau expression was observed with a slight higher Tau level in *S305N* Tau cells compared to WT and P301S cell lines (Fig. 1B).

### Analysis of Tau phosphorylation in differentiated SH-SY5Y cells

Phosphorylation was monitored first by SDS-PAGE and immunoblotting using anti-phospho-tau antibodies. Results showed no significant alteration in tau phosphorylation among the different cell lines at commonly studied AD deregulated phosphoepitopes such as AT180, AT270, PHF-1 and 12E8 [13] (data not shown).

**Figure 2:**
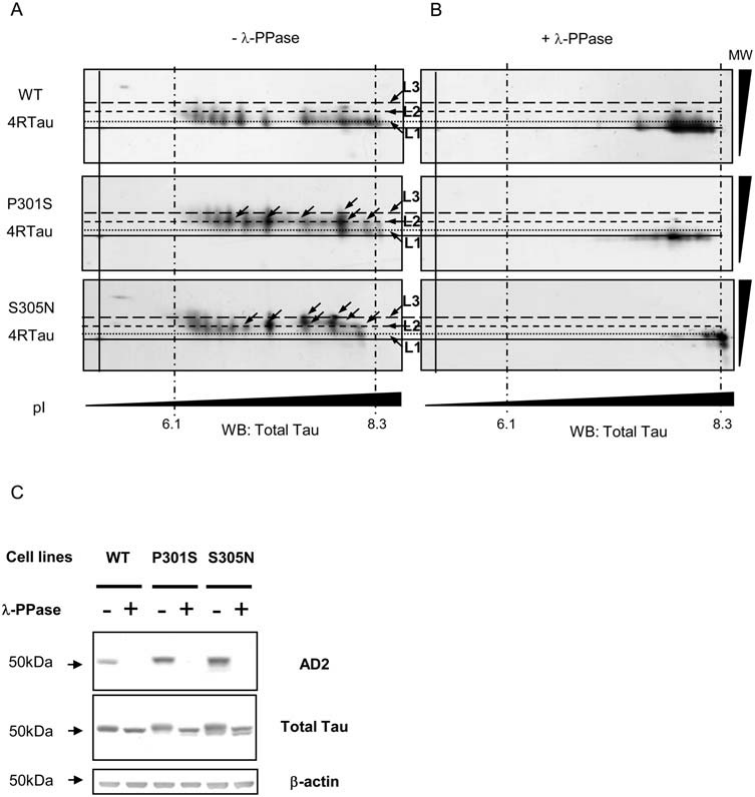
Analysis of Tau phosphorylation in 4RTau cell lines by 2D electrophoresis. A) Bi-dimensional analysis of *WT* and Mutated *P301S* or *S305N* 4RTau. Differentiated cells treated with tetracycline for 48 hours were subjected to 2D analysis and immunoblotted with Tau Exon10 antibody (4RTau specific). Alignments of immunoblots were performed using unspecific products revealed by secondary antibody. Different molecular weight (MW) levels (levels1–3: L1–L3) of Tau isovariants are represented by short dashed lines and pI indicators (obtained with an internal IEF control) by long and short mixed dashed lines. B) Bi-dimensional analysis of *WT* and Mutated 4RTau after *in vitro* Tau dephosphorylation followed by immunoblotting with Tau exon10 antibody. C) Immunoblot analysis of *in vitro* dephosphorylated *WT* and mutated (*P301S* and *S305N*) 4RTau. Differentiated cells treated with tetracycline for 48 hours were subjected (+) or not (−), to an *in vitro* dephosphorylation by λ-phosphatase and immunoblotted by phosphorylation dependent antibody. Total amount of Tau proteins loaded is visualized with TauCter 1902 antibody (Total Tau) and β-actin was used as loading control.

To investigate overall Tau phosphorylation state, bidimensional (2D) electrophoresis was performed, followed by an immunostaining with Tau-exon10 antibody to reveal total exogenous 4RTau (Fig. 2A). Analysis revealed about 10 species of Tau isovariants that are displayed, for both *WT* and mutated 4RTau, on three levels of apparent molecular weight (L1–L3). Interestingly, while *WT* 4RTau isovariants are displayed over a wide range of isoelectric point (pI) at mainly L1 molecular weight, isovariants for *P301S* and *S305N* mutations are found proportionally at less acidic pI and at higher molecular weight (L2 and L3). In fact, *P301S* and *S305N* isovariants within the basic pI range displayed a molecular weight shift. This shift, which is characterised by an increase in isovariants number on high molecular weight levels (Fig. 2A: black arrows, levels 2 (L2) an 3 (L3)) is indicative of a significant difference in electrophoretic mobility between *WT* and mutated 4RTau consistent with phosphorylation [14]. To determine whether such difference is linked to Tau phosphorylation status, we performed *in vitro* Tau dephosphorylation by λ-phosphatase prior to 2D electrophoresis. Dephosphorylation efficiency was first verified by SDS-PAGE followed by immunoblotting with antibodies against phospho-Tau. As shown in Fig. 2C, a complete dephosphorylation at AD2 epitope was observed after treatment with λ-phosphatase. Besides that, immunoblot with a total Tau antibody revealed a higher electrophoretic mobility visualized as a thin “down shift” of mutated 4RTau treated with λ-phosphatase (compared to untreated ones). This “down shift” is also observed for dephosphorylated *WT* 4RTau, but to a lesser extent.

Analysis of dephosphorylated Tau by 2D-electrophoresis and immunoblotting with Tau exon10 antibody showed that all isovariants for both *WT* and mutated 4RTau are displaced into the most basic pI range (Fig. 2B). More importantly, we observed that the molecular weight “shift” of mutated 4RTau isovariants is completely abolished after treatment by λ-phosphatase. Altogether, these data clearly support that, compared to *WT* 4RTau, mutated 4RTau likely display a difference in phosphorylation pattern which is responsible for a substantial molecular weight “shift”.

### Overall Tau phosphorylation analysis in transgenic mice

**Figure 3:**
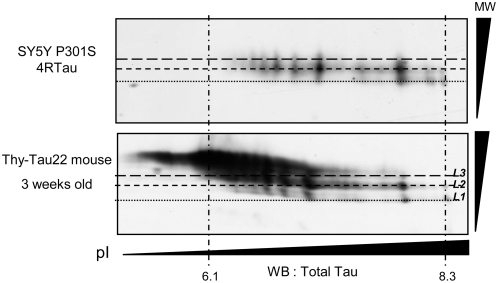
Analysis of overall Tau phosphorylation in THY-Tau 22 transgenic mice. Comparison of 2D analysis of human exogenous Tau in 3-weeks old Thy-Tau22 trangenic mice and mutated *P301S* 4RTau from SY5Y Cells. Alignments of immunoblots were performed using unspecific products revealed by secondary antibody. Different molecular weight (MW) levels (L1–L3) of Tau isovariants are represented by short dashed lines and pI indicators (obtained with an internal IEF control) by long and short mixed dashed lines. Exogenous Tau in mice was probed by an anti-human Tau antibody and exogenous *P301S* 4RTau in SY5Y cells was visualized by Tau-Exon10 antibody.

To analyse relevance of the findings described above, 2D analysis of Tau was performed in a recent characterized transgenic mouse model relevant to Tau pathology in Alzheimer's disease [1]. These mice displayed a quite well defined neurofibrillary degeneration time course, which is mainly characterized by Tau hyperphosphorylation, abnormal Tau phosphorylation and appearance of neuronal inclusions with “paired helical filaments” structures between 6 to 10 months. These Tau abnormalities are associated to spatial memory impairments. Analysis of overall Tau phosphorylation by 2D analysis followed by immunoblots with antibodies specifically directed against Total exogenous human Tau revealed the presence of Tau isovariants in a pI range with high molecular weight levels (L2 and L3) consistent with the pattern observed for mutated Tau in SH-SY5Y cell lines (Fig. 3). This analysis performed in brains of 3 weeks old mice suggest that this molecular event appears very early in the degeneration process.

### Tau aggregation analysis by electronic microscopy

**Figure 4:**
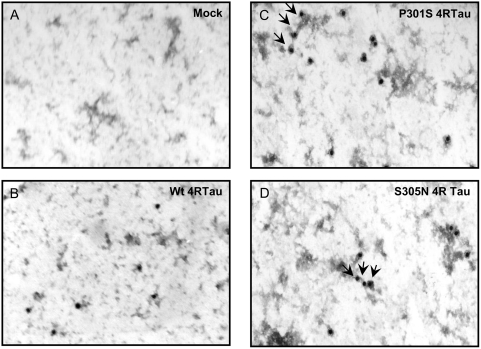
Analysis of Tau aggregation by electronic microscopy and Gold-immunolabeling. Lysates from differentiated Mock and 4RTau cell lines, treated with tetracycline for 48 hours, were subjected to immunogold labelling with TauCter 1902 (Total Tau) antibody and revealed by colloidal gold labelled secondary antibody. Images were obtained at high magnification: 20 000×. “Clustering” of Tau immunoreactivity is indicated by black arrows.

As in transgenic mice the specific pattern of Tau phosphorylation precedes aggregation, human neuronal cell lines were analyzed for Tau aggregation. Lysates from differentiated cells were hence analysed by immunogold labeling electron microscopy. Results showed a Tau labelling, which is “clustered” on opaque structures in mutant 4RTau cell lines (Fig. 4C and D: black arrows). These structures are specific to mutated 4RTau cell lines since they are never observed neither in Mock or *WT* 4RTau cell lines (Fig. 4A and B). Neverthelesss, as reported in previous cellular models, these structures are still far different from the main typical Tau aggregates found in Tauopathies since they are neither straight nor paired helical filaments.

## Discussion

Here, we reported a new model that allows an inducible over-expression of either *WT* or mutated 4RTau bearing FTDP-17 mutations. This model may be appropriate to investigate molecular events which could trigger Tau fibrillogenesis in a “neuronal like” cell context. Indeed, electron microscopy analysis in our cell models showed no Tau aggregates similar to those observed in transgenic mice models or pathological human brains. It confirms results from a long list of previous cellular models, which have never clearly shown the existence of such filaments. However, as described in neuroglioma H4 cells [15], our cell lines display some Tau clustering structures which may correspond to very early starting points of Tau nucleation. These data likely suggest that some additional factors could be involved in Tau fibrillogenesis and then would be required to trigger the whole tangle pathology.

In the present study, Tau phosphorylation was analysed by 2D electrophoresis. FTDP-17 mutated 4RTau were found differentially phosphorylated compared to *WT* 4RTau. Within the basic pI range, the number of Tau isovariants with a L1 electrophoretic mobility is higher in WT 4RTau than mutated Tau. More importantly, 4RTau mutants isovariants showed a distinct 2D-migration pattern characterised by a molecular weight shift (towards L2 and L3), which is abolished after *in vitro* dephosphorylation. This “shift” could be due to specific mode of Tau phosphorylation so called “state I” described by Lindwall and col. [16]. In this study, authors suggested, as described before for Histone H1 [17], that Tau proteins contain domains resistant to SDS which remain under a folded state and migrate rapidly by electrophoresis. Phosphorylation of a cluster of epitopes located in these domains or their vicinity could lead to conformational changes and hence allowing appearance of “stiffer” Tau forms, which have a lower electrophoretic mobility. Moreover, it may be hypothesized that these changes are not related to an increase in Tau phosphorylation of commonly studied AD epitopes since our analysis by classical SDS-PAGE revealed neither an increase nor a decrease of phosphorylation at these epitopes in 4RTau mutants. These data are coherent with previous studies, which similarly report no significant change in Tau phosphorylation (for review [11]). In fact, only rare FTDP-17 mutations such as R406W lead to a decrease in Tau phosphorylation [18], [19]. Finally, in order to investigate the *in vivo* relevance of the molecular shift displayed in mutated 4RTau cell lines, we performed the same overall approach with a Tau transgenic mice model, which exhibits a quite well defined Tau pathology associated to memory deficits [1]. Interestingly, we observed the same Tau phosphorylation pattern as in Tau mutants cell lines. More importantly, this molecular shift is observed in 3-weeks old mice, before the main features of Tau pathology, defined by the appearance of abnormal and hyperphosphorylated Tau aggregates, that occurs in 6 to 10 months-old mice.

In summary, 2D approach reveals that *P301S* and *S305N* 4RTau mutants exist under a particular phosphorylation state. Our data suggest that pathological Tau mutations may both change distribution of phosphate groups and generate “clusters of phosphorylation”. This work could raise a common mechanism for several FTDP-17 mutations, which could act through the dysregulation of Tau phosphorylation status ultimately leading to conformational changes. Finally, our data underline the possibility that this molecular event could be one of the first Tau modifications during the neurofibrillary degeneration cascade since this phenomenon appears prior to Tau pathology in an *in vivo* model and is linked to early step of Tau nucleation in Tau mutants cell lines. Such cell lines consist in suitable and evolving models to investigate additional factors involved in molecular pathways leading to whole Tau aggregation.

## Materials and Methods

### cDNA constructs

The Tau cDNA coding for the human 412 amino acids 4-Repeat (4R) isoform was a kind gift from M. Goedert (Medical Research Council Laboratory of Molecular Biology, Cambridge, UK). A polymerase chain reaction (PCR)-based site-directed mutagenesis (QuickChange, STRATAGENE, The Netherlands) was used to generate two distinct constructs coding for the 4RTau *P301S* or the 4RTau *S305N* mutants. Wild-type and mutated cDNAs were sequenced and inserted in the tetracycline inducible mammalian expression T-REX vector pcDNA4/TO © (INVITROGEN, France).

### Cell Culture and stable transfections

Generation of human SH-SY5Y cell line overexpressing tetracycline repressor has been described elsewhere [20]. This cell line was used for stable transfection of either pcDNA4/TO empty vector or pcDNA4/TO containing Tau cDNAs (*WT* or mutated). For differentiation, cells were maintained for 7 days in DMEM/F12 medium supplemented with 2 mM L-glutamine, 50 U/mL penicillin/streptomycin, 7 µg/mL progesterone, 1% Insulin/Transferrin/selenium (INVITROGEN) and 5 ng/ml β-NGF (REPROTECH INC., TEBU, France) [21], [22]. Medium was replenished every 3 days. To induce transgenes expression, cells are maintained in medium with 1 µg/ml tetracycline [20].

### Electron microscopy and Gold-immunolabeling

Cells were harvested in MES buffer (0.1 M MES, 2 mM MgCl_2_, 0.5 M EGTA, 1 mM NaCl, pH 6.5) with complete protease inhibitors (ROCHE APPLIED SCIENCE, France) and phosphatase inhibitor: 125 nM okadaic acid (SIGMA-ALDRICH, France). Cells were lysed using a dounce homogenizer and then collected on perlodion coated nickel-200 mesh grids. After 10 mn blocking with 1% gelatine-PBS at room Temperature, grids were incubated with TauCter 1902 antibody according to Table 1. After washing, colloidal gold (18 nm) labelled-goat anti-rabbit immunoglobulins were used. Finally, counterstaining was made in 4% uranyl acetate in H_2_O. Observations were done with a Zeiss 9025 electron microscope.

**Table 1:** Antibodies used in immunoblotting studies.

Antibody	Species	Specificity	Dilution	References
AD2	Mouse	Tau; pSer396-pS404	1∶20 000*	[14]
M19G	Rabbit	Tau; human specific	1∶20 000*	[14]
TauCter 1902	Rabbit	Total Tau	1∶4 000*	[24]
Tau-Exon10 (E10)	Rabbit	Total 4RTau	1∶4 000*	[25]
β-actin	Mouse		1∶50 000*	Sigma-Aldrich

* Blocking: Tris-buffered saline, pH 8, 0.05% Tween 20+5% skim Milk.

### Western Blotting

Cells and mouse brain tissues were processed as previously described [1], [20]. Total proteins concentration was determined by the BCA assay Kit (PIERCE, Perbio, France). Proteins were mixed to LDS Sample Buffer containing a reducing agent (INVITROGEN NP-009) and boiled at 100°C for 10 minutes, as recommended by the manufacturer (INVITROGEN). For SDS-PAGE, 10 µg of total proteins were loaded onto a 10% acrylamide gel (Novex NuPAGE® INVITROGEN), blotted onto nitrocellulose or polyvinylidene difluoride membranes (Hybond and Hybond-Phosphate from AMERSHAM/GE HEALTHCARE, France). Membranes were blocked and incubated with the appropriate antibody according to Table 1 and then incubated with a Horseradish peroxidase-conjugated secondary antibody (Goat anti-rabbit A4914 from SIGMA-ALDRICH and Horse anti-mouse from Vector Laboratories). Finally, peroxidase activity was revealed with the ECL detection kit and visualized with Hyperfilm™ ECL™ (AMERSHAM/GE HEALTHCARE).

For two-Dimensional (2D) gel electrophoresis 100 µg of total proteins were dissolved in 400 µl of 2D electrophoresis buffer (7 M urea, 2 M thiourea, 4% (v/v) Triton X-100, 20 mM dithithreitol and 0.6% (v/v) pharmalytes). Samples were loaded on immobilized pH gradient ReadyStrip IPG strip 3–10 (BIORAD, France), isoelectrofocused with the Protean IEF cell (BIORAD) by applying a total of 75 kV/h, according to the manufacturer's instructions (For each experiment, samples are processed in the same run of isoelectrofocalisation). The IPG strips were then equilibrated three times (15 mn each) in a Laemmli Buffer (25 mM TrisHCl, 20 mM DTT, 10% Glycérol, 5% SDS, 0.05% Bromophenol Blue, pH 6.8) and were layered onto a 10% Tris-Glycine Polyacrylamide Gel. SDS-PAGE was performed with a Protean II Xi Cell (BIORAD) and blotted onto nitrocellulose or polyvinylidene difluoride membranes with Criterion Blotter (BIORAD) as recommended by the manufacturer. Membranes were then incubated with either anti-E10tau antibody or anti human tau antibody (Table 1).

### Tau dephosphorylation In Vitro

Cells were harvested into an ice-cold NP-40 lysis buffer (50 mM Tris-HCl, pH 7.4, 150 mM NaCl, 1 mM EDTA, 0.1% NP-40) supplemented with protease inhibitors. Amount of total proteins was determined using the BCA kit and 100 µg of total proteins, with protease inhibitors to avoid any protein degradation, were incubated at 37°C, for 80 minutes with λ-protein phosphatase (EC 3.1.3.16) in reaction buffer (2 mM MgCl_2_, NEB reaction buffer 1×) as recommended by the manufacturer (New England Biolabs, Ozyme, France) [23]. Dephosphorylation was stopped on ice and samples were immediately mixed to LDS Sample Buffer for SDS-PAGE or dissolved in a 2D electrophoresis buffer for 2D analysis, as described above.
